# Engineering selection stringency on expression vector for the production of recombinant human alpha1-antitrypsin using Chinese Hamster Ovary cells

**DOI:** 10.1186/1753-6561-9-S9-P10

**Published:** 2015-12-14

**Authors:** Christine L Chin, Hing K Chin, Cara S Hui Chin, Ethan T Lai, Say KongNg

**Affiliations:** 1 Bioprocessing Technology Institute, Agency for Science, Technology and Research (A*STAR), Singapore; 2 Department of Pharmacy, Faculty of Science, National University of Singapore, Singapore

## Background

Currently, the titers of biopharmaceutical production from Chinese hamster ovary (CHO) cells have achieved gram per liter range and this can be attributed to advances in bioprocess development, media development and cell line development. To obtain high producing cell lines, extensive screening of producer clones during cell line development is often necessary. To improve the efficiency and efficacy of generating and isolating high producing clones, various expression vector engineering technologies can be applied, for example ubiquitous chromatin opening element (UCOE) [1], matrix attachment regions (MARs) [2](Mirkovitch et al. 1984), site specific recombination [3-9], to improve selection stringency [10-13], and to co-localize the GOI with the selection marker [14-16].

In our previous studies, we have similarly shown that specific productivities can be improved when we increased selection stringency by destabilizing the selection marker through the addition of AU-rich elements (ARE) to promote mRNA degradation and murine ornithine decarboxylase (MODC) PEST region to enhance protein degradation [17]. While coexpression of GOI and selection marker using multiple promoters on the same vector may help in the co-localization, we have previously demonstrated that gene fragmentation can occurs at a high level of 14% during stable transfection of dual promoter dicistronic vector in CHO-DG44 cells [18]. Subsequently, an attenuated IRES element was used together with the PEST region to allow for high recombinant protein titer using stably amplified cell pools [19].

In this study, we evaluated the use of tandem PEST sequence, further attenuation of the IRES element, and codon-deoptimization of the dhfr selection marker, to further optimizing the strength of selection marker expression in CHO cells for the production of recombinant human Alpha1-antitrypsin (rhA1AT), a serum protease inhibitor currently purified from human blood plasma as replacement therapy for patients who developed chronic obstructive pulmonary disease due to deficiency in the protein. Such vector combinations to attenuate translation initiation, protein elongation and protein stability for optimizing selection stringency have not been previously investigated. To our knowledge, there is also no report on high-titer production of rhA1AT in CHO cells, which is necessary for its manufacturability due to its high dosage requirement.

## Experimental approach

**Figure 1:**
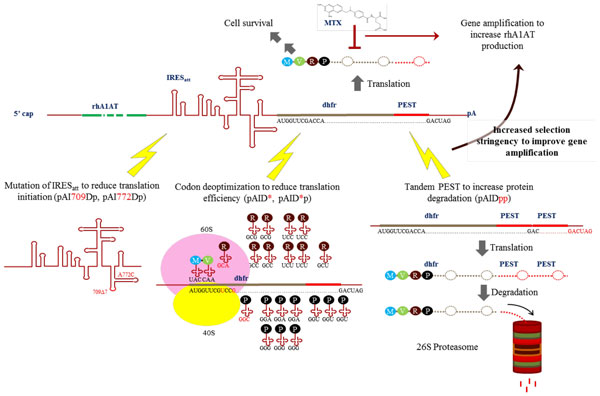
**Strategies for selection marker attenuation**.

7 expression vectors expressing rhA1AT that can be classified into 3 sets (Figure 1) were designed. Using rhA1AT as the gene of interest, the first vector set consists of pAID, pAIDp and pAIDpp. Comparing data from the use of pAIDp against pAID will allow us to validate the use of PEST element in improving stable recombinant gene expression, as observed in our previous studies [17,19]. The application of 2 tandem PEST elements in pAIDpp then allowed us to determine whether an additional PEST can further improve stable recombinant gene expression, as this has not been demonstrated in literature to our knowledge. The second vector set consists of pAI709Dp and pAI772Dp. These 2 vectors incorporated mutations described by Hoffman MA and Palmenberg AC [20] into the attenuated IRES [21,22]. This is to evaluate whether the further attenuation of selection marker expression with these additional impediment in translation initiation can improve stable recombinant gene expression. The third vector set comprised of pAID* and pAID*p. These 2 vectors incorporated a codon de-optimized dhfr selection marker to evaluate the use of codon deoptimization as a strategy to further reduce selection marker expression levels, since it will theoretically reduce translation efficiency, a different aspect of gene expression that is not affected by the attenuated IRES and PEST. The selection and amplification efficiency, recombinant protein productivity, relative transcript copy numbers and dhfr expression levels were then analyzed.

## Results and discussion

pAIDpp and pAI772Dp vectors gave further improvements in rhA1AT production when compared to pAID and pAIDp vectors, indicating that further selection marker attenuation can improve recombinant protein production. Using the pAI772Dp vector, we generated a cell pool that gave a maximum titer of 1.05 g/l of rhA1AT in an un-optimized shake flask batch culture using a 2-step amplification till 50 nM MTX that took less than 3 months (Table 1). Using the pAI772Dp and pAIDpp vectors, we generated cell pools that gave a maximum titer of 1.11 and 1.15 g/l respectively in un-optimized shake flask batch cultures at 300 nM MTX (Table 1). To our knowledge, this is the highest reported recombinant protein titer from shake flask cultures of stable mammalian cell pools.

**Table 1:** Growth and productivity of top cell pools.

MTX concentration	Vector	Pool 1				Pool 2				Average		
		Max titer^1 ^(mg/l)	q_p_ (pcd)	t_D _(h)	Fold titer increase ^2^	Max titer^1 ^(mg/l)	q_p_ (pcd)	t_D _(h)	Fold titer increase ^2^	Max titer^1 ^(mg/l)	q_p_ (pcd)	t_D _(h)
**50 nM**	pAID	256	17.6	46	5.6					256	17.6	46
	pAIDp	492	25.7	36	4.2	244	10.9	36	3.1	368	18.3	36
	pAIDpp	647	29.0	33	4.4	539	25.9	44	5.4	593	27.5	39
	pAI772Dp	1054	41.3	28	8.5	937	33.8	26	8.2	996	37.6	27
	pAID*	275	14.4	41	3.1	170	7.8	40	2.2	222	11.1	40
	pAID*p	277	8.4	33	2.0					277	8.4	33
**300 nM**	pAID	560	32.5	34	2.2					560	32.5	34
	pAIDp	514	25.8	33	1.0	240	13.1	37	1.0	377	19.5	35
	pAIDpp	1146	88.2	45	1.8	846	41.9	41	1.6	996	65.0	43
	pAI772Dp	1111	48.6	42	1.1	863	35.4	27	0.9	987	42.0	34
	pAID*	721	34.3	43	2.6	398	22.1	33	2.3	559	28.2	38
	pAID*p	412	17.7	29	1.5					412	17.7	29

^1 ^Maximum titer was taken as the highest rhA1AT titer assayed from the first 11 days of the batch culture.

^2 ^Fold titer increase for cell pools in 50 nM MTX suspension culture was compared against the titers of the same cell pools in adherent 96 well plate cultures, whereas that for cell pools in 300 nM MTX suspension culture was compared against the titers of the same cell pools in 50 nM MTX suspension culture.

Relative transcript copy numbers demonstrated that the transcription of rhA1AT and dhfr genes were correlated due to the IRES linkage, although the results also suggested that the protein expression were not solely dependent on transcript levels. Protein level analysis of dhfr validated that the cell pools were indeed expressing the modified dhfr of the correct molecular weight. In addition, it showed that the strategies of further attenuating dhfr protein expression with the use of a mutated IRES and tandem PEST, but not codon deoptimization, were effective in reducing dhfr protein levels in these MTX amplified cell pools in suspension serum free culture. Our data suggests the codon usage of surviving cells with codon deoptimized selection marker may be changed in our culture conditions to enable better cell survivability. Hence, this result suggest that codon usage of the selection marker should be considered for applications that involve gene amplification and serum free suspension culture, since the general expression and regulation of host cell proteins may be affected due to a change in codon usage in the surviving cells.
